# Prognostic factors of worse outcome for hospitalized COVID-19 patients, with emphasis on chest computed tomography data: a retrospective study

**DOI:** 10.31744/einstein_journal/2022AO6953

**Published:** 2022-05-16

**Authors:** Adham do Amaral e Castro, Patrícia Yokoo, Eduardo Kaiser Ururahy Nunes Fonseca, Jessyca Couto Otoni, Sarah Lustosa Haiek, Hamilton Shoji, Rodrigo Caruso Chate, Andrea Z Pereira, Marcos Roberto Gomes de Queiroz, Marcelo Costa Batista, Gilberto Szarf

**Affiliations:** 1 Hospital Israelita Albert Einstein São Paulo SP Brazil Hospital Israelita Albert Einstein, São Paulo, SP, Brazil.; 2 Hospital Israelita Albert Einstein Goiânia GO Brazil Hospital Israelita Albert Einstein, Goiânia, GO, Brazil.

**Keywords:** Coronavirus infections, COVID-19, Pneumonia, Obesity, Multidetector computed tomography, Tomography, X-ray computed, Prognosis

## Abstract

**Objective:**

To evaluate anthropometric and clinical data, muscle mass, subcutaneous fat, spine bone mineral density, extent of acute pulmonary disease related to COVID-19, quantification of pulmonary emphysema, coronary calcium, and hepatic steatosis using chest computed tomography of hospitalized patients with confirmed diagnosis of COVID-19 pneumonia and verify its association with disease severity.

**Methods:**

A total of 123 adults hospitalized due to COVID-19 pneumonia were enrolled in the present study, which evaluated the anthropometric, clinical and chest computed tomography data (pectoral and paravertebral muscle area and density, subcutaneous fat, thoracic vertebral bodies density, degree of pulmonary involvement by disease, coronary calcium quantification, liver attenuation measurement) and their association with poorer prognosis characterized through a combined outcome of intubation and mechanical ventilation, need of intensive care unit, and death.

**Results:**

Age (p=0.013), body mass index (p=0.009), lymphopenia (p=0.034), and degree of pulmonary involvement of COVID-19 pneumonia (p<0.001) were associated with poor prognosis. Extent of pulmonary involvement by COVID-19 pneumonia had an odds ratio of 1,329 for a poor prognosis and a cutoff value of 6.5 for increased risk, with a sensitivity of 64.9% and specificity of 67.1%.

**Conclusion:**

The present study found an association of high body mass index, older age, extent of pulmonary involvement by COVID-19, and lymphopenia with severity of COVID-19 pneumonia in hospitalized patients.

## INTRODUCTION

Since December 2019, the world has been experiencing the COVID-19 pandemic.^(1)^ Imaging findings of COVID-19 pneumonia involve ground glass opacities, crazy paving and consolidations - usually bilateral, with subpleural and peripheral distribution, most often affecting the pulmonary bases.^(2)^

Clinical and laboratory factors associated with COVID-19 pneumonia severity, such as advanced age, comorbidities, including obesity, elevated D-dimer levels, and lymphopenia are already known.^(2,3)^ Imaging findings related to the most severe forms of the disease include greater extent of lung involvement and increased rate of lung opacities.^(2)^

In other clinical scenarios, many factors measurable on chest computed tomography (CT) are associated with worse outcomes, such as muscle mass,^(4)^ coronary calcium,^(5)^ chronic obstructive pulmonary disease,^(6)^ osteoporosis, vertebral fractures,^(7-9)^ and hepatic steatosis.^(10)^

So far, there are few studies that explored the analysis of data obtained through a chest CT in the context of pneumonia by COVID-19 and no Brazilian or Latin American experience with this study design, which is the contribution of the present study.

## OBJECTIVE

To evaluate the association of anthropometric, clinical, laboratory, and some potentially useful measurable factors on chest computed tomography (pectorals and paravertebral muscle mass, subcutaneous fat, spine bone mineral density, extent of acute pulmonary disease of COVID-19, coronary calcium, pulmonary emphysema and hepatic steatosis) with worse outcome (reflected by the need of mechanical ventilation or intensive care, or death) in a group of hospitalized patients with COVID-19 pneumonia.

## METHODS

### Patients

This study was approved by the Research Ethics Committee of the *Hospital Israelita Albert Einstein* (HIAE), # 4.445.142, CAAE: 30810420.4.0000.0071. Informed consent was waived due to its retrospective nature.

The participants of the present study were patients aged over 18 years, who underwent hospital admission for pneumonia caused by COVID-19, confirmed by the real-time reverse transcription polymerase chain reaction (RT-PCR) method,^(11)^ and who also underwent a chest CT scan during the first day of hospital admission between March 1^st^ and April 31^th^, 2020, totaling 123 patients.

### Anthropometric, clinical, and laboratory data

The anthropometric, clinical, and laboratory data evaluated at hospital admission were sex, age, body mass index (BMI), referred days since symptoms onset and admission to the emergency room, oxygen saturation (SpO_2_), days of hospital stay, blood count, C-reactive protein, creatinine, lactic acid dehydrogenase (LDH), D-dimer, interleukin-6, ferritin, corticotherapy use, presence of deep vein thrombosis, pulmonary embolism, and lymphopenia.

### Computed tomography

Computed tomography scans were acquired using multidetector CT scanners with 40, 80 or 320 detector rows (Biograph mCT, Siemens Healthcare, Erlangen, Germany or Aquilion Prime or Aquilion ONE, Canon Medical Systems, Tochigi, Japan). Regarding the acquisition parameters, the scans were obtained according to institutional protocol, in the supine position during end-inspiration, with or without intravenous contrast material, reconstructed slice thickness of 1mm, voltage of 80-120kVp, and automatic milliampere setting ranging from 10 to 440mA. All the CT scans were performed during the first day of hospital admission.

The following analyses were performed, as previously described in the literature: pectoral and paravertebral muscle mass and subcutaneous fat,^(4)^ bone mineral density,^(9)^ presence of vertebral body fractures,^(12,13)^ degree of pulmonary involvement by COVID-19 pneumonia,^(14)^ quantification of pulmonary emphysema,^(15)^ coronary artery calcium quantification,^(5)^ and hepatic steatosis.^(10)^ These measurements are described in more detail in the supplementary material.

### Anthropometric measurement

The patients’ weight (kg) was determined using a calibrated scale. They were barefoot and wearing light clothing. Their height (m) was measured with the use of a stadiometer with patient standing barefoot, feet together, arms outstretched beside the body, and straight back.

Body mass index was calculated with weight (kg) divided by the squared height (m), and used to classify nutritional status of the adult patients as: <16kg/m^2^: malnutrition grade III; 16-16.9kg/m^2^: malnutrition grade II; 17-18.4kg/m^2^: malnutrition grade I; 18.5-24.9kg/m^2^: normal; 25-29.9kg/m^2^: overweight; 30-34.9kg/m^2^: obesity class I; 35-39.9kg/m^2^: obesity class II; ≥40kg/m^2^: obesity class III.^(16)^

### Statistical analysis

Patients’ age was described using summary measures (mean, standard deviation, median, minimum, and maximum).

Qualitative characteristics were described according to worse outcome (need of mechanical ventilation or intensive care, or death). Absolute and relative frequencies and the association with the use of *χ*^2^ or exact tests (Fisher’s exact test or likelihood-ratio test) were used. Quantitative characteristics were described according to the same outcome. Summary measures were used and compared between the outcome, using the Mann-Whitney test.

The multiple logistic regression model was adjusted for worse outcome, inserting the statistically significant and clinically relevant variables in the model, and using the stepwise backward method (with 5% entry and exit criteria as the selection criterion).

Interreader agreement was determined by calculating intraclass correlation coefficient (ICC). Interreader agreement was considered to be poor (0.20), fair (0.21-0.40), moderate (0.41-0.60), good (0.61-0.80), or excellent (0.81-1.00).

IBM-SPSS for Windows version 22.0 software was used to perform the analyzes and Microsoft Excel 2010 software was used to tabulate the data. The tests were performed with a significance level of 5%.

## RESULTS

A total of 123 patients were included in this study. The mean (SD) age was 57.4 (+16.5) years, 79 (64.2%) were men, and the mean BMI was 27.9 (+ 5.1) kg/m^2^.

The median days of hospital stay was 11, ranging from 1 to 117 days. The median days from symptoms onset to CT imaging was 6, ranging from 1 to 21 days. Thirty-eight (30.9%) patients were exclusively in a ward bed, while 47 (38.2%) needed a semi-intensive care unit bed, and 38 (30.9%) needed an intensive care unit bed. Thirty-five patients (28.5%) needed orotracheal intubation with mechanical ventilation (MV), with an average length of 6.3 days (+14 days). A total of 6 patients (8.6%) died. The baseline characteristics are summarized in table 1.

**Table 1:** Baseline clinical and laboratorial characteristics of the patients included in the study

Characteristic		Worse outcome MV/ICU/Death (n=38)	Better outcome No MV/ICU/Death (n=85)	p value
Age		65.2+14.2	53.9+16.4	<0.001†
Sex				0.291†
	Women	11 (28.9)	33 (38.8)	
	Men	27 (71.1)	52 (61.2)	
BMI (kg/m^2^)		29.6±6.4	27.2±4.2	0.038†
Time from symptom onset (days)		6.3±4.7	6.7±3.2	0.697†
SpO_2_		92.1±4.8	94.9±2.9	0.002*
Hospital length of stay (days)		41.8±28.7	10.1±9.5	<0.001*
Creatinine (mg/dL)		1.2±0.8	1.1±1.0	0.34†
C-reactive protein (mg/dL)		138.8±112.3	60.4±57.0	<0.001*
Red blood cell (count/mm^3^)		4.36 ± 0.8	4.7 ± 0.5	0.009†
Lactate dehydrogenase (U/L)		360.1±131.9	285.4±113.8	0.003*
D-dimer (ng/mL)		1,338.1±1,874.3	794.1±1,089.6	0.017*
Ferritin (ng/mL)		2,187.8±2,120.4	1,140.1±1,131.8	0.013*
Interleukin 6		710.4±1,359.8	65.4±89.8	0.003*
Corticotherapy		30 (78.9)	21 (24.7)	<0.001†
Deep vein thrombosis		6 (15.8)	1 (1.2)	0.003*
Pulmonary embolism		6 (15.8)	0 (0)	0.001*
Lymphopenia		25 (65.8)	35 (41.2)	0.012†
Obesity		16 (42.1)	20 (23.5)	0.036†

Results expressed as n (%), or mean±standard deviation.

* Fisher’s exact test;

† *χ*^2^ test.

SpO_2_: oxygen saturation; MV: mechanical ventilation; ICU: intensive care unit; BMI: body mass index; Lymphopenia <1,000 count/mm^3^; 30 – 34.9kg/m^2^: obesity = BMI ≥30.0.

Regarding CT parameters, interreader agreement was excellent for all measures (0.81-1.00), except for the mean density of the pectoral muscle, which was good (0.61-0.80). The CT parameters are summarized in table 2.

**Table 2:** Computed tomography characteristics of the patients included in the study

Characteristic	Worse outcome MV/ICU/Death (n=38)	Better outcome No MV/ICU/Death (n=85)	p value
Total pectoral area (pectoral sum) (cm^2^)	49.3±57.1	43.7±19.7	0.420†
Average pectoral density (HU)	32.6±12.4	37.5±12.3	0.042†
Pectoral index (total pectoral area/height²)	16.3±18.5	14.1±5.1	0.309†
Total subcutaneous (subcutaneous sum) (cm^2^)	61.7±36.8	54.1±29.2	0.223†
Subcutaneous index (total subcutaneous area/height²)	20.9±13.5	18.4±11.1	0.289†
Total paravertebral (paravertebral sum) (cm^2^)	33.1±8.4	31.8±10.2	0.510†
Average paravertebral density (HU)	35.1±17.2	40.7±13.4	0.078†
Paravertebral index (total paravertebral area/height²)	11.0±10.4	10.4±2.7	0.213†
Average bone densities (HU)	163.5±42.6	200.0±55.3	<0.001†
Vertebral body fracture	3 (7.9)	4 (4.7)	0.675†
Degree of pulmonary involvement	11.7±6.9	6.3±2.5	<0.001*
Coronary calcium	22 (57.9)	31 (36.5)	0.027†
Pulmonary emphysema	4 (37.5)	6 (25)	0.668†
Average liver attenuation	48.3±11.7	52.7±10.9	0.047†
Hepatic steatosis	9 (23.7)	8 (9.4)	0.034†

Results expressed as n (%), or mean±standard deviation.

* Fisher’s exact test;

† *χ*^2^ test.

HU: Hounsfield unit; MV: mechanical ventilation; ICU; intensive care unit; Degree of pulmonary involvement – range from 0 to 24; coronary calcium – presence in any degree.

After logistic regression, age, BMI, degree of pulmonary involvement, and lymphopenia were identified as factors independently associated with worse outcome (Table 3).

**Table 3:** Variables associated with worse outcome of the patients included in the study

Worse outcome MV/ICU/Death (n=38)	OR	95%CI	p value*
Age	1.042	1.009-1.076	0.013
BMI (kg/m^2^)	1.157	1.157-1.037	0.009
Degree of pulmonary involvement	1.329	1.158-1.526	<0.001
Lymphopenia	3.084	1.091-8.72	0.034

* *χ*^2^ test.

MV: mechanical ventilation; ICU: intensive care unit; BMI: body mass index; Lymphopenia <1,000 count/mm^3^; OR: odds ratio; 95%CI: 95% confidence interval.

**Figure 1:**
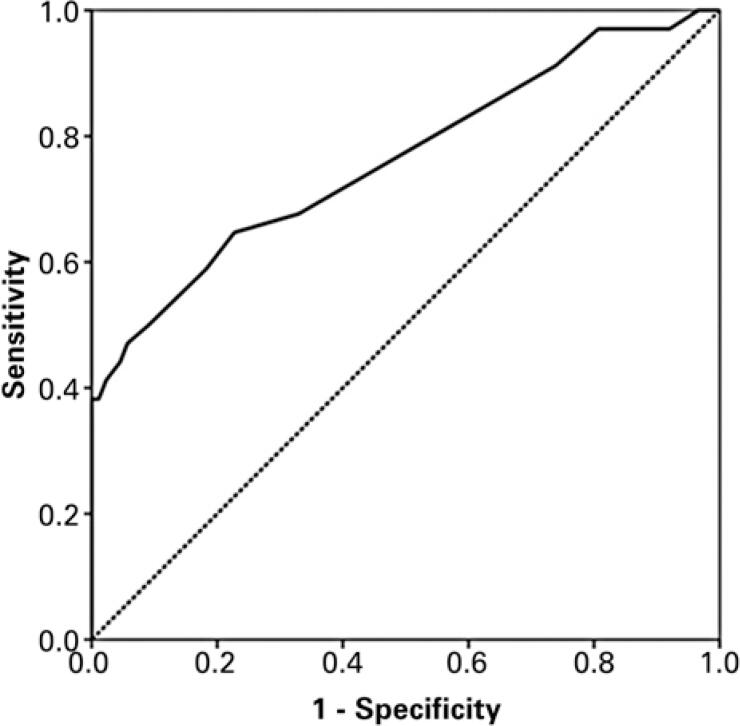
Receiver operating characteristic curve of visual score of disease extent as an indicator of poorer prognosis (mechanical ventilation/intensive care unit/death) due to COVID-19 pneumonia. The area under the receiver operating characteristic curve was 0.764 (95%CI: 0.66-0.86)

More extensive pulmonary involvement by COVID-19 pneumonia was associated with worse outcome (MV/ICU/death) with the best cutoff point for the visual score of disease extent of 6.5, with a sensitivity of 64.9% and specificity of 67.1%, which can be observed in figure 1.

## DISCUSSION

The outcomes of patients hospitalized for COVID-19 pneumonia were assessed through measurement of a combined outcome of intubation and MV, need of ICU, and death. The data analyzed were those commonly requested and available in patients diagnosed with COVID-19 giving emphasis to chest CT.

In a meta-analysis, Hussain et al. demonstrated higher mortality due to COVID-19 pneumonia in patients older than 70 years, BMI >25kg/m^2^ and advanced respiratory support need.^(3)^ The present study also showed greater severity of the disease in relation to age and higher BMI. Hendren et al. observed worse prognosis for patients with obesity (death and MV), especially in the youngest (under 50 years old). One of the possible explanations was a weaker association of BMI in older individuals and the presence of other causes of morbidities and fragility.^(17)^

When assessing patients’ obesity, it is particularly important to remember that this is not usually an isolated condition, but a manifestation of a complex metabolic syndrome, in which the patient is in a pro-inflammatory state, related to multiple negative outcomes. Liver steatosis is one of the most important conditions within the systemic metabolic disorders triggered by obesity and is highly related to an increase in BMI and visceral fat. Although the present study did not find an association between steatosis and a worse outcome of COVID-19 pneumonia, Medeiros et al. showed a higher frequency of hepatic steatosis in patients diagnosed with COVID-19 pneumonia, but they did not access its relationship with the severity of the disease.^(18)^

The use of bone density data in routine chest CT scans has been evidenced in the literature, having a good correlation with bone mineral density measured by dual-energy X-ray absorptiometry (DEXA), the gold standard for that evaluation.^(19,20)^ Although this study did not find an association between bone mineral density and worse outcomes, the access to bone mineral density in chest CT can provide relevant information for the clinician as for the medical state that can be used to predict a worse prognosis. This information was not explored extensively in COVID-19 patients, one exception being the study of Ji et al. which demonstrated an association of the diagnosis of COVID-19 with osteoporosis, but was not associated with the severity of the disease.^(21)^

This study did not find an association between pulmonary emphysema and worse prognosis. These findings differ from the study by Zhang et al. who observed an association of deaths from COVID-19 with pulmonary emphysema.^(22)^ This can be explained perhaps by a selection bias of this sample (analysis of hospitalized patients only, that is, more complex disease configuring worse prognosis by definition) or even by the low number of deaths.

Coronary artery calcium is a factor known to be associated with cardiovascular risk in asymptomatic adults and with overall mortality. Regarding the severity of patients with COVID-19, the present study did not demonstrate an association of coronary calcium with worse prognosis. Ferrante et al. evaluated risk factors for myocardial injury and death in patients with COVID-19, coronary calcium was also not shown to be an independent risk factor.^(23)^

Regarding the pulmonary findings of COVID-19 pneumonia, studies indicate some imaging characteristics that may be related to worse prognosis, as in the study by Li et al., who observed an association of consolidation, linear opacities, crazy-paving pattern, bronchial wall thickening, high CT scores, and extrapulmonary lesions being features of severe/critical COVID-19 pneumonia.^(2)^ In the study by Tabatabaei et al. severity score was the only statistically significant CT predictor of mortality in patients.^(24)^ These findings are in agreement with the present results, because this study also observed an association of the degree of pulmonary involvement with disease severity (need for intubation and MV and the total days of intubation). This study found an optimal cutoff value of a CT score of 6.5 (with a sensitivity of 64.9% and specificity of 67.1%) to predict the need for intubation and MV. Yuan et al. already suggested an optimal cutoff of 24.5 (sensitivity of 85.6% and specificity of 84.5%) for the prediction of mortality considering the lung parenchyma CT score with a different methodology, with values ranging from 0 to 72.^(25)^

Schiaffino et al. also studied chest CT-derived muscle status of patients hospitalized for pneumonia due to COVID-19 and differently from this study, they found an independent association of low muscle mass with ICU admission and mortality.^(26)^ Kim et al. also investigated the association of sarcopenia with the outcomes of patients hospitalized for pneumonia due to COVID-19 using chest CT data, with a single-center sample, similar in size to the present study,^(27)^ and they stratified patients according to muscle mass and found an association between low muscle mass and longer hospital stay, but, as in this study, low muscle mass was not an independent risk factor for death.^(27)^ These data show the importance of a larger sample and multicentric based data to identify the association of muscle mass with worse outcomes, demonstrated in the study by Schiaffino et al.^(26)^

The present study also identified an association of lymphopenia with worse outcome of the disease. These data are in agreement with the meta-analysis by Zhao et al. which also demonstrated an increased risk of severe disease, with OR=2.99, 95%CI: 1.31-6.82.^(28)^ The mechanisms for this reduction in lymphocyte count is not fully elucidated, but as discussed by Tan et al. it may involve factors such as direct viral infection, destruction of lymphatic organs, production of inflammatory cytokines, and inhibition of production by metabolic disorders such as hyperlactic acidemia.^(29)^ Although this study did not perform a dynamic analysis of the variation of this lymphopenia throughout the research, this information, which is extremely available in the emergency room, can make the clinician alert in cases of newly admitted patients with lymphopenia.

Some limitations of the present study are its retrospective design and the evaluation of more severe patients, that is, those in which a therapeutic decision of hospitalization has already been made. Also, although CT was performed on the day of hospital admission, the time between symptom onset among patients was different. Other limitations and the main ones are the low number of patients, single-center basis, and low number of deaths. Also, DEXA, which is considered the gold standard for body quantification, was not used. Besides that, visceral and parietal fat was not analyzed as the study did not have abdominal CT data.

The present study demonstrated that data easily obtained, such as older age, higher BMI, lymphopenia, and high pulmonary involvement, may be associated with worse outcome in patients hospitalized for COVID-19 pneumonia. This can potentially be included in clinical scores and eventually help to predict which patients should be monitored for worse progression or even more aggressive therapeutic decisions.

## CONCLUSION

The present study found an association of higher body mass index, older age, extent of pulmonary involvement by COVID-19, and lymphopenia with the severity of COVID-19 pneumonia in hospitalized patients.
